# Cumulative culture can emerge from collective intelligence in animal groups

**DOI:** 10.1038/ncomms15049

**Published:** 2017-04-18

**Authors:** Takao Sasaki, Dora Biro

**Affiliations:** 1 Department of Zoology, University of Oxford, South Parks Road, Oxford OX1 3PS, UK

## Abstract

Studies of collective intelligence in animal groups typically overlook potential improvement through learning. Although knowledge accumulation is recognized as a major advantage of group living within the framework of Cumulative Cultural Evolution (CCE), the interplay between CCE and collective intelligence has remained unexplored. Here, we use homing pigeons to investigate whether the repeated removal and replacement of individuals in experimental groups (a key method in testing for CCE) alters the groups' solution efficiency over successive generations. Homing performance improves continuously over generations, and later-generation groups eventually outperform both solo individuals and fixed-membership groups. Homing routes are more similar in consecutive generations within the same chains than between chains, indicating cross-generational knowledge transfer. Our findings thus show that collective intelligence in animal groups can accumulate progressive modifications over time. Furthermore, our results satisfy the main criteria for CCE and suggest potential mechanisms for CCE that do not rely on complex cognition.

## Abstract (web summary)

Groups of animals tend to solve tasks better than individuals, but it is unclear whether such socially-derived knowledge accumulates over time. Sasaki and Biro demonstrate that homing pigeon flocks progressively improve the efficiency of their routes by culturally accumulating knowledge across generations.

Across many animal taxa, individuals form groups that collectively process information and make joint decisions^1^,^2^. By pooling information, these groups can generate better decisions than solitary agents—a phenomenon referred to as collective intelligence^3^. To date, studies of collective intelligence have typically focused on one-off performance, such as a swarm of bees choosing to settle at one of several available nest sites^4^ or a group of jurors reaching a verdict in court^5^, and have generally assumed that collective decisions are not influenced by information acquired from past experiences involving similar scenarios.

However, because animal groups in nature often face the same tasks repeatedly, feedback from past outcomes has the potential to influence future behaviour and decision quality^6^,^7^. Such ‘collective learning'^8^,^9^ allows individuals to acquire valuable information through acting collectively with others, and may provide crucial input when the same task is undertaken by the group again. Iterative solving of a given task may thus lead to the accumulation of knowledge within the group, and in turn improve collective performance over time^10^,^11^.

Improvements in behavioural solutions to specific social, life history or ecological problems are also central to the study of culture^12^. The accumulation of knowledge through individual invention and subsequent social learning has been recognized as an important advantage of group living. In addition, culture can accumulate progressively, or ‘ratchet', over generations—a process referred to as Cumulative Cultural Evolution (CCE)^12^,^13^,^14^,^15^. CCE can allow groups to develop increasingly complex knowledge and skills over time, beyond the capacities of a single individual. Although CCE is a well-established research field, the interplay between CCE and collective intelligence has so far remained unexplored.

In the present study, our aim was to investigate the cumulative potential of collective intelligence—and hence its capacity to support CCE—by devising a scenario in which we can systematically quantify both the appearance of novel innovations that build on existing behaviours^16^ and their cross-generational maintenance in real time.

We used collective navigation by homing pigeons, *Columba livia*, as our model system, examining whether collective intelligence undergoes cumulative changes over time in an ecologically relevant, recurring collective problem-solving scenario. The system has proved valuable in studies of collective decision-making^9^,^17^ because pigeons have been shown to be able to process information both individually and collectively: previous work using miniature global positioning system (GPS) tracking has revealed that (i) given sufficient experience, individual birds develop idiosyncratic homing routes, which they recapitulate faithfully^18^ and (ii) flocks can also collectively develop distinctive routes^10^, including those obtained by the averaging of individual preferences^17^. These features allowed us to directly compare ‘solutions' consisting of stable and distinctive routes between individuals and groups.

Furthermore, route information can be passed on through social learning^10^. Interestingly, although naive ‘observer' pigeons closely follow experienced leaders^19^, they remain active participants in the route-finding process, frequently changing, indeed improving, the efficiency of their leaders' routes^10^. Thus, we hypothesized that these ‘innovations'—existing routes altered then learnt by newly added individuals—present an opportunity for progressive improvement in collective homing performance over time.

For our design, we adapted methods developed for testing CCE in human subjects^20^: generational succession was simulated through the sequential replacement of experienced birds with naive birds within 10 independent chains as they were repeatedly required to solve the same (navigational) task. The first ‘generation' in each chain consisted of a single individual, which, once it developed a stable homing route, was paired and flown with a naive partner; then in each pair experienced birds were replaced by further naive birds in stages, to create subsequent generations of pairs (Fig. 1). As our control treatments, we released 10 additional birds individually and 20 additional birds as fixed pairs (that is, 10 pairs, with consistent pair membership), for the same total number of releases as performed by our chains of pairs.

Our data show that, through building on knowledge transferred across generations, homing performance in the experimental group improved continuously, eventually outperforming both control groups. Therefore, our results not only confirm that collective intelligence can become a cumulative process in animal groups, but, by satisfying the main criteria for CCE, they also demonstrate the presence of CCE in a non-human species.

## Results

### Route efficiency improvement

**Figure 2:**
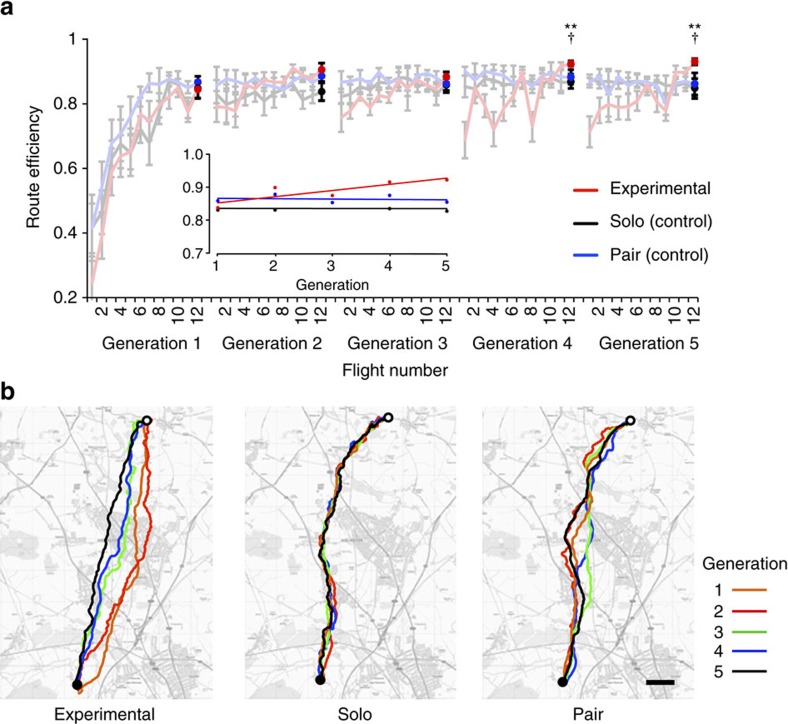
Changes in homing efficiency over five generations. (**a**) Homing efficiency for all flights in the experimental group (red), the solo control group (black) and the fixed-pair control group (blue). The final (twelfth) flight of each generation in the experimental group and the equivalent (twelfth, twenty-fourth, thirty-sixth, forty-eighth and sixtieth) flights in the control groups are highlighted as bold symbols. Mann–Whitney–Wilcoxon test: **P<0.01 and ^†^*P*<0.05, for the solo versus experimental comparison and for the fixed-pair versus experimental comparison, respectively. Error bars show s.e.m. Inset shows linear mixed-effects model fitted to the final flights of each generation for all three treatment groups. (**b**) Examples of route development in the three treatment groups. The left panel shows final flights performed by each generation (first–fifth) in one chain in the experimental group; middle and right panels show the equivalent flights performed by a solo control bird and a fixed-pair control, respectively. The release point is indicated by a white dot, the home loft by a black dot. Scale bar represents 1 km. Map image: OS data © Crown copyright and database right (2017).

Birds in all three treatment groups improved steadily in homing efficiency (measured as the beeline distance between release and home, 8.6 km, divided by the length of the path flown) over the first 12 flights (Fig. 2a). The performance of single birds in the solo control group and in the first generation of the experimental group did not differ significantly on the twelfth flight (Mann–Whitney-–Wilcoxon test: *W*=49, *P*=0.78)—this was expected, since they had received identical treatment up to this point—nor did the performance of the control (fixed) pairs from either solo controls (*W*=24, *P*=0.78) or first-generation experimental birds (*W*=34, *P*=0.71).

In the control groups, where birds continued to fly as solos or fixed pairs for the rest of the experiment, efficiency plateaued around the thirteenth flight, once both solos and pairs had established and began to recapitulate their idiosyncratic routes (Fig. 2a and Supplementary Fig. 1; consistent with previous findings^9^,^10^). In the experimental group, whenever naive partners were added to experienced birds (that is, to ones that had flown the route previously) at the start of each generation, we observed an initial drop in the efficiency of the pair, but in each case this recovered as flight number increased within the given generation. Indeed, each generation typically outperformed, eventually, the previous generation's peak efficiency (Page's trend test: *L*=399, *k*=5, *n*=8, *P*<0.01; Fig. 2a).

To test for differences in homing efficiency between the experimental group and the two control groups over the full course of releases, we compared the last (twelfth) flight of each generation in the experimental group with the equivalent flights (twelfth, twenty-fourth, thirty-sixth, forty-eighth and sixtieth) in the control groups (Fig. 2a). The efficiency of pairs in the experimental group continued to improve over generations, with routes flown at the end of the fifth generation 1.2 km shorter than those at the end of the first generation. In contrast, the solo control group shortened their routes by only 0.05 km and the pair control group extended theirs by 0.04 km over the same period. Indeed, changes in route efficiency progressed at different rates in the three treatment groups (linear mixed-effects model with efficiency as the dependent variable, treatment group, generation number and their interaction as fixed effects, and bird/pair identity nested within treatment group as a random effect: *χ*^2^=4.59, df=1, *P*=0.032), where the slope of the experimental group was significantly different from those of either of the control groups (*t*=2.10, df=112, *P*=0.038 and *t*=2.00, df=112, *P*=0.047 for solo versus experimental and pair versus experimental, respectively; see the inset in Fig. 2a). Pairwise comparisons within each generation further showed that the experimental group outperformed both solo and paired controls by the fourth generation and beyond (Mann–Whitney–Wilcoxon test: fourth generation: *W*=65, *P*=0.003, distance difference (*d*_exp-solo_)=0.67 km and *W*=45, *P*=0.035, *d*_exp-pair_=0.55 km; fifth generation: *W*=60, *P*=0.002, *d*_exp-solo_=0.99 km and *W*=42, *P*=0.019, *d*_exp-pair_=0.87 km for solo versus experimental and pair versus experimental, respectively; Fig. 2a). In sum, successions of pairs improved in performance over generations and reached better performance than solo individuals or fixed pairs who had the same number of flights.

### Transfer of route information across generations

**Figure 3:**
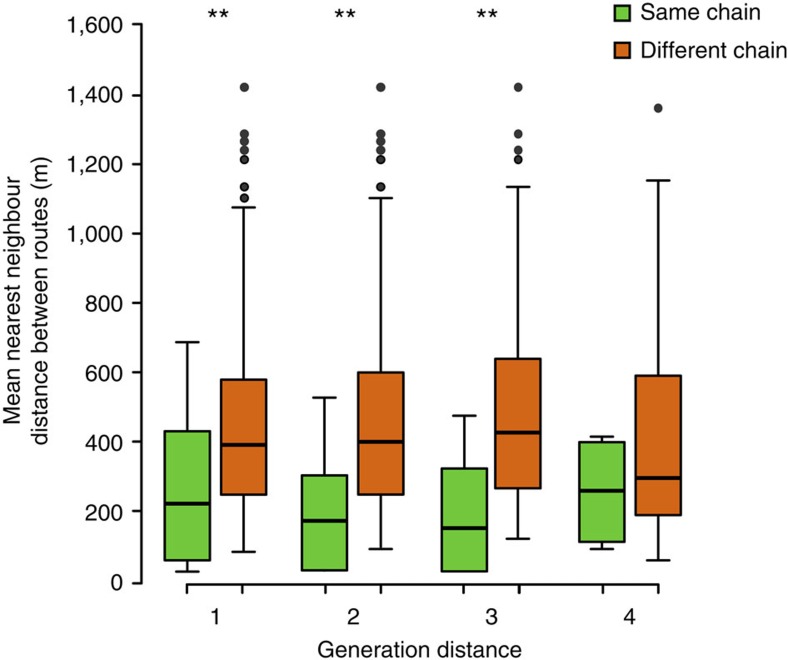
Route similarities within and between chains of generations in the experimental group. Mean nearest-neighbour distances (see Methods) are plotted between flights within the same chain and between different chains in the experimental group, at all possible generation distances (that is, differences in ordinal generation numbers). Larger values indicate lower route similarities. Each box extends between the lower and upper quartiles, the horizontal line within the box corresponds to the median and whiskers show the range of the data, except for outliers indicated by circles. Mann–Whitney–Wilcoxon test: **P<0.01.

To quantify the transfer of route information between individuals and between generations, we calculated route similarities within the same chain and between different chains in the experimental group. Given that routes generated by solo birds are known to be idiosyncratic and to differ between individuals^18^, if social transmission was operating within chains then we expected that routes would show higher similarity within the same chain than between different chains. This held true for distances of up to three generations (Mann–Whitney–Wilcoxon test: *W*=3,378, *P*<0.001; *W*=1,334, *P*<0.001; and *W*=420, *P*<0.001, for generation distances 1, 2 and 3, respectively; Fig. 3). For flights separated by four generations, routes in the same chain were no longer more similar to each other than they were to routes in different chains (*W*=189, *P*=0.11). This was likely because, as generation distance increased, not only were there fewer potential comparisons to be made (reducing statistical power) but also the routes analysed necessarily included later-stage ones: as these all typically converged on the beeline, they homogenized variation across chains (Figs 2b and 3).

## Discussion

Our results satisfy, through systematic analysis, the main criteria for CCE^13^,^20^. First, we showed that homing performance improved over consecutive ‘generations' of pairs, consistent with a ‘ratchet effect'^15^. Second, pairs at the end of generational succession outperformed solo individuals^12^ as well as pairs without turnover in membership. Finally, these solutions (homing routes) were more similar in consecutive generations within the same chains than between chains, indicating that knowledge was transferred across generations^20^.

That chains of pairs outperformed not only solos but also fixed pairs indicates that the collective improvement was not simply a consequence of the averaging of ‘many wrongs'^21^, or of ‘two heads being better than one'. Indeed, solos and control pairs reached similar asymptotes in their performance after approximately 13 releases, once their idiosyncratic routes had been established. In contrast, the chains of pairs in the experimental group continued to improve—a phenomenon unexpected under current theories of route navigation in pigeons^22^.

We propose that cumulative improvement was exhibited in our navigational problem-solving scenario because (a) information pooling allows birds introduced in each new generation to contribute novel ‘innovations' that can outperform previous solutions^10^, (b) pigeons are capable of learning solutions produced through collective intelligence (‘collective learning'^8^,^9^) and use these as inputs in subsequent collective decisions and (c) pigeons are capable of evaluating the payoffs of their performance, such that when errors do get added by naive individuals, these innovations can be ‘pruned' on the basis that they lead to worse performance (while those that lead to better routes are kept). Notably, the filtering process highlighted in (c) is also recognized as an important mechanism in CCE^23^,^24^. The combination of these phenomena adds a hitherto little explored time-depth perspective^11^—the notion that current performance is contingent on a previous history of performances—to collective behaviour through empirical demonstration, and introduces an important conceptual link between collective intelligence and CCE. CCE is frequently argued to be unique to humans, and this uniqueness is typically attributed to the convergence of complex cognitive scaffolds (such as prosociality, teaching and high-fidelity imitation) that are thought to support the phenomenon^25^. Although recent studies have identified the purported existence of CCE in non-humans^26^,^27^, evidence remains inconclusive in the absence of data on the ancestral and intermediate states of the behaviours in question. Our results, on the other hand, directly document the process of progressive modification, and reveal potential mechanisms for CCE that do not rely on complex cognition. Indeed, because debates over sufficient and necessary mechanisms for CCE frequently centre around social learning mechanisms^13^,^14^,^25^,^28^,^29^, future work should examine the precise nature of social transmission operating during route learning in pigeons.

Two key differences between our study and the commonly used interpretation of CCE are that (i) we demonstrated increase in efficiency but not in complexity and (ii) our task had a well-defined end point representing maximal efficiency (the beeline path) rather than being open-ended. Although both increasing complexity and open-endedness have undoubtedly played major parts in the immense technological, social and communicative sophistication that has emerged in human behaviour^15^, the accumulation of knowledge over generations—exceeding the capacities of single individuals (and, in our case, also of static groups)—remains a fundamental aspect of our results and demonstrates a more widespread potential for the phenomenon than previously thought.

In summary, we have demonstrated that, by facing the same problem repeatedly, collective intelligence in animal groups can become a cumulative process. We would predict similar accumulation of knowledge in other multi-generational social groups, ranging from the establishment of ‘traditional' foraging areas in social insects^30^ to long-distance migration routes in whooping cranes^31^. Our results complement mathematical models suggesting that CCE is dependent on demography^32^, where, in addition to emphasizing populations as pools of potential social learners and rare innovators, we also emphasize that they represent pools of information from which innovations emerge through collective intelligence. Further, our inferences are in line with recent laboratory studies of CCE in humans that demonstrate important benefits to allowing different, rather than always the same, group members to contribute to collective performance^24^. We anticipate that future research both on humans and on non-humans will integrate the fields of collective intelligence and CCE more closely, and elucidate the circumstances under which even agents with limited cognitive abilities can make progressively superior decisions over time.

## Methods

### Subjects and experimental protocols

We used 60 homing pigeons, *Columba livia*, bred at the Oxford University Field Station, Wytham, UK (51°46′58.34″N, 1°19′02.40″W). Subjects were between 2 and 9 years of age and had participated in experiments in previous years, but had never been released from the vicinity of the current release site. All were equipped with an elastic harness ‘backpack' carrying a plasticine dummy weight (15 g). Dummy weights were replaced by GPS loggers (see below) during all experimental releases.

All releases were conducted from the same site, Greenhill Farm (51°51′23.8″N, 1°17′03.0″W; distance to home: 8.6 km, direction to home: 197°). The experimental group consisted of 10 independent chains of ‘generations' as follows. In the first generation of each chain, a single pigeon was released individually 12 times (a number shown by previous research to be sufficient to allow birds to develop stable routes^10^,^18^). We then paired each first-generation pigeon with a naive individual (a bird that had never previously visited the site), and released them together a further 12 times (second generation). Thereafter the original first-generation bird was replaced by a new naive bird and this new pair was also released 12 times (third generation). This procedure was repeated until five generations were tested in each of the 10 independent chains. In the two control groups, 10 solo pigeons and 10 pairs (20 pigeons, with pair membership constant throughout the experiment) were released the same total number of times (60) as that experienced by the five experimental generations. Up to two releases were conducted per day, with a minimum of 1 h between releases, in dry weather and at wind speeds <10 ms^−1^.

One experimental pair in the fourth generation (and the subsequent pair in the fifth generation) and another in the fifth generation were excluded from analysis because the birds split up during all 12 of their flights (splitting was defined as individuals released in a pair becoming separated by more than 150 m at any point during flight^10^). Additionally, one bird in the solo control group did not return home on its thirteenth release. In the fixed pair control group, three birds belonging to different pairs did not return home on their first release. We also excluded a pair that split up in more than 90% of their total flights. Thus, the sample size for the experimental group was 9 in the fourth generation and 8 in the fifth generation, for the solo control group it was 9 from the thirteenth release onwards and for the fixed pair control group it was 6 from the first release.

### Data logging

Flight tracks of individual birds were recorded using 5 Hz GPS loggers (15 g; BT-Q1300ST, Qstarz, Taiwan) and downloaded to a computer using Qtravel (Qstarz) software. Tracks were analysed in MATLAB (MathWorks, 2011), after converting raw positional data from degrees to metres using a Universal Transverse Mercator grid. In order to focus on the choice of homing route, segments of track within a 200 m radius of the loft were excluded (here, birds typically circled around the loft immediately prior to landing). Route efficiency and route similarity measures were extracted from flight data using established methods (for example, ref. 17; see the next section for details of calculations).

### Data analysis

In total, 1,080 flights were recorded in the experimental group, 552 in the solo control group and 840 in the pair control group. Of these, 59 (5.4%), 22 (3.9%) and 31 (3.6%), respectively, failed to provide full track data due to GPS device failure. These flights are therefore missing from the analysis. In addition, on 22, 5 and 4 occasions, respectively, birds took longer than the device's battery life to return home, and thus we obtained only partial GPS tracks from these flights. For our analysis, we assumed that these birds flew directly home from the location where they were at the time the GPS battery ran out; we therefore underestimated their routes as their actual routes were most likely considerably longer. In all three groups, these occasions were confined only to the first 3–4 releases of any given bird (that is, within generations in the case of the experimental group). Because we did not use data from early flights in our comparisons of route efficiency and route similarity between groups, any bias we might have introduced into these flights by estimating their missing portions had no effect on our overall conclusions.

To compare homing performance between birds in the control and experimental groups, we extracted two measures from each flight's GPS data: route efficiency and route similarity. Route efficiency was calculated by dividing the direct straight-line distance from the release point to home by the actual distance flown. Shorter routes therefore corresponded to higher efficiency values (approaching the maximum of 1). Route similarity was measured as the mean nearest-neighbour distance between a focal track and a chosen reference track. For each point on the focal track, the distance to the closest point on the reference track (for example, a previous flight) was measured, and the mean of these distances was calculated. Lower mean nearest-neighbor distances therefore corresponded to greater similarities between tracks.

A given pair's route efficiency in the experimental group and in the fixed pair control group was calculated as the mean of the two birds' efficiencies. Similarly, a given pair's route similarity to its previous flight was calculated as the mean of the two birds' nearest-neighbour distances to their own respective previous routes.

Route efficiency was first compared among the three different treatment groups using a linear mixed-effects model (inset in Fig. 2a). The model included route efficiency as the dependent variable, treatment group, generation number and their interaction as fixed effects, and bird/pair identity nested within treatment group as a random effect. *Post hoc* pairwise comparisons were conducted using Mann–Whitney–Wilcoxon tests (Fig. 2). Page's *L* Trend Test^33^ was used to test successive improvement over generations.

Route similarity was compared between the control pair and solo groups by linear models (Supplementary Fig. 1):


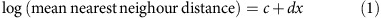


where *x* is the number of release, *c* is the intercept and *d* is the slope. The mean nearest-neighbour distances between releases were log-transformed as these distances were highly skewed. We tested the null hypothesis that the parameter *c* did not differ between the solo and pair control groups using the same technique as described above.

When assessing the transfer of route information between generations, we calculated route similarities within the same chain and between different chains at all possible generation distances, from 1 (that is, consecutive) to 4 (that is, between the first and the fifth generation), and between all possible pairings of birds, excepting any that actually flew together. In other words, the latter rule meant that for consecutive-generation comparisons within the same chain, we compared the track of the ‘old' (that is, experienced) bird in a given generation with that of the ‘new' (that is, naive) bird in the next generation. One exception to this was the comparison of the first and the second generation because the first generation did not have ‘old' birds. Even excluding these data, routes were significantly more similar within the same chain than between different chains for generation distances of 1 (Mann–Whitney–Wilcoxon test: *W*=2,264, *P*<0.01). We used the last flight of each generation to represent birds' routes in these comparisons, because routes were typically established by then (Supplementary Fig. 2). The similarities were compared using Mann–Whitney–Wilcoxon tests as they were not normally distributed (Fig. 3). The statistical package R (v. 3.2.1) was used for all analyses.

### Data availability

All the data (GPS tracks of all flights in the experiment) are available from T.S. upon request.

## Additional information

**How to cite this article:** Sasaki, T. & Biro, D. Cumulative culture can emerge from collective intelligence in animal groups. *Nat. Commun.* **8,** 15049 doi: 10.1038/ncomms15049 (2017).

**Publisher's note:** Springer Nature remains neutral with regard to jurisdictional claims in published maps and institutional affiliations.

## Supplementary Material

Supplementary Information Supplementary Figures

## Figures and Tables

**Figure 1:**
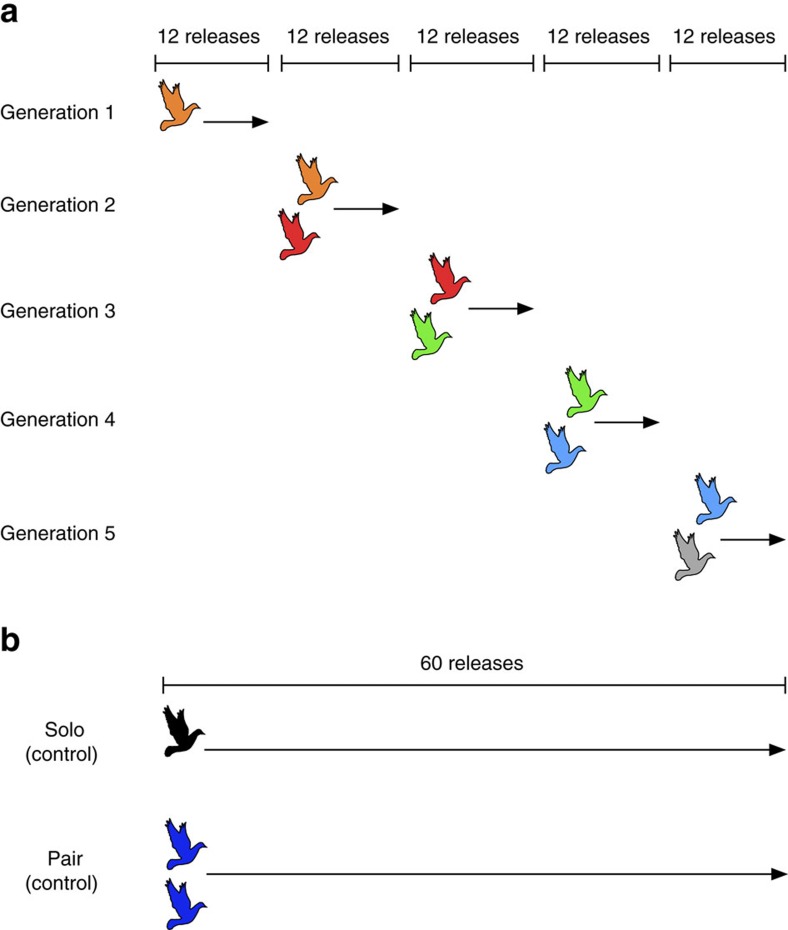
Homing flight release protocols. (**a**) Experimental group; (**b**) control groups. In each chain of the experimental group, a single pigeon (orange) was first released from the same site repeatedly 12 times, then partnered with a naive pigeon (red) and flown as a pair a further 12 times. The first bird was then replaced by a third bird (green) and this new pair (red+green) was also released 12 times. This procedure continued until the fifth-generation bird (grey) was added and flown a final 12 times. In the control groups (**b**), single pigeons and fixed pairs were released the same number of times as the total flown by the experimental group (60 flights). All three treatment groups contained 10 independent replicates (chains, solo birds or pairs).
