# Supplementary material for: Cumulative culture can emerge from collective intelligence in animal groups
Source: Nat Commun. 2017 Apr 18;8:15049. doi: 10.1038/ncomms15049 (PMC5399285; doi:10.1038/ncomms15049)
Supplement: Supplementary Information — Supplementary Figures [file ncomms15049-s1.pdf]

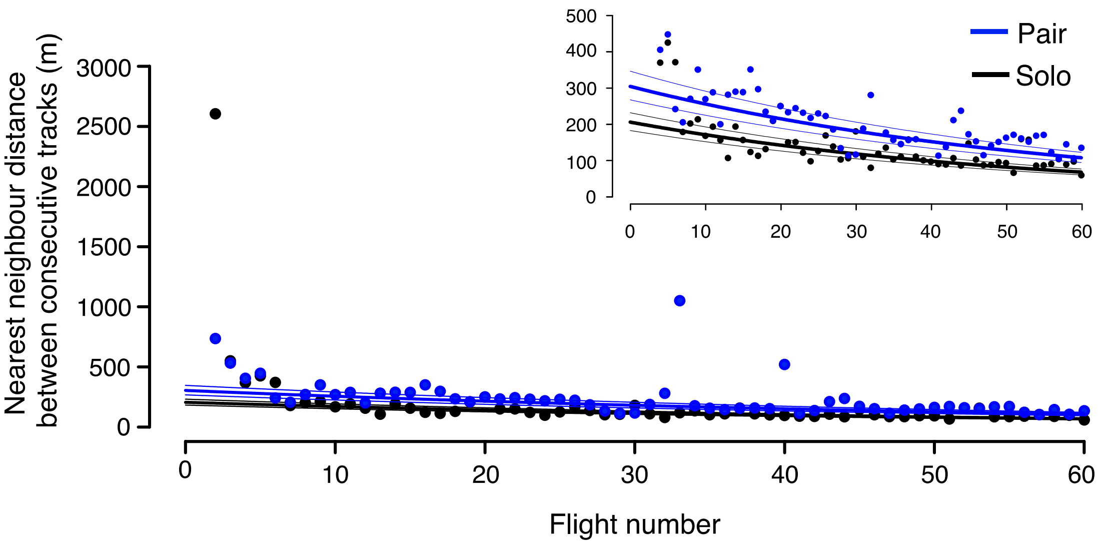

Supplementary Figure 1 | Mean nearest neighbour (NN) distance between consecutive tracks in the solo and pair control groups. NN distance is calculated as the spatial proximity between consecutive tracks flown by a given bird (see Supplementary Information for more detail). Mean NN distance decreases with increasing flight number in both groups, showing that through training individual birds and pairs gradually settle on a consistent route they subsequently continue to recapitulate. However, the asymptotic level of mean NN distance is higher for the pair group (blue line) than for the solo control group (black line; $p < 0.01$), showing that pairs recapitulate their routes less accurately than individual birds. Thick lines represent exponential decay functions fitted to each group's data (see Supplementary Information for details of the analysis). Thin lines show 95% confidence intervals. The inset in the top right corner is a magnified version (i.e. the y-axis is constrained to 0 - 500) of the original graph for better visibility of the fitted lines.

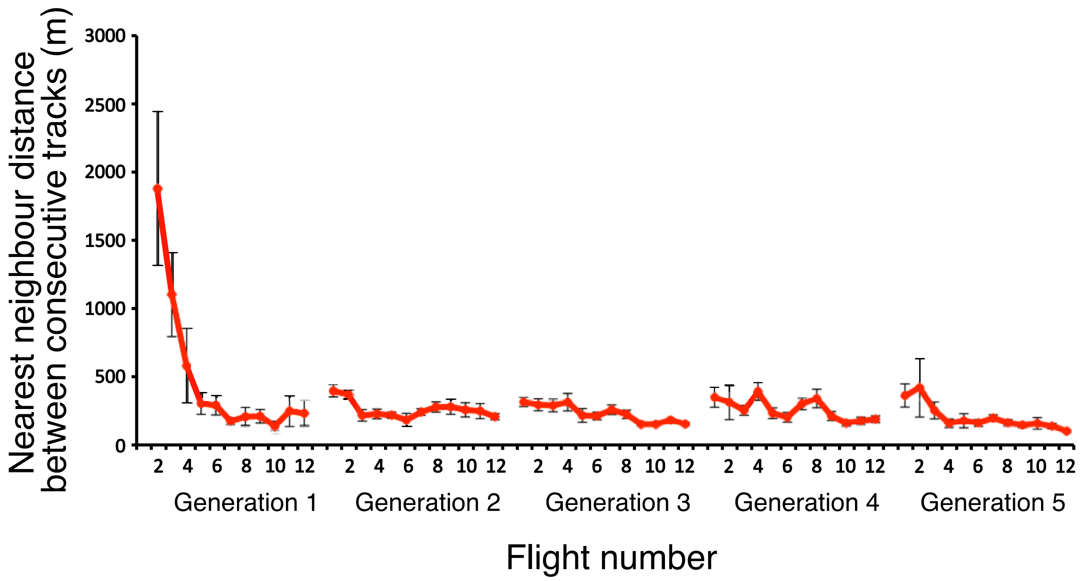

Supplementary Figure 2 | Mean nearest neighbour (NN) distances between consecutive tracks in the experimental group. NN distance is calculated as the spatial proximity between consecutive tracks flown by a given bird (see Supplementary Information for more detail). Mean NN distance decreases with increasing flight number in each generation, showing that pairs gradually settle on a consistent route they subsequently continue to recapitulate. Note that at the start of each generation there is an increase in mean NN distance to the previous flight (final flight of the previous generation), indicating that the introduction of a naïve bird shifted the pair away from the previously established route. Solid symbols show means across the 10 independent transmission chains (except in the 4th and 5th generations, which had 9 and 8 chains, respectively); error bars show s.e.m.
